# Multi-omics analysis reveals coordinated transcriptional and epigenetic remodeling of tenocytes under IL-1β-driven inflammatory stimulation

**DOI:** 10.3389/fimmu.2026.1940588

**Published:** 2026-10-19

**Authors:** Wenyan Zhou, Xiaonan Wang, Junxin Lin, Zaixing Yang

**Affiliations:** 1 Department of Laboratory Medicine, Taizhou First People’s Hospital (Taizhou University Affiliated First People’s Hospital), School of Medicine, Taizhou University, Taizhou, Zhejiang, China; 2 School of Medicine, Taizhou University, Taizhou, Zhejiang, China

**Keywords:** ATAC-seq, IL-1β, multi-omics, RNA-seq, tenocyte

## Abstract

**Background:**

Tendinopathy is frequently associated with chronic inflammation driven by pro-inflammatory cytokines. Among these, interleukin-1β (IL-1β) serves as a central mediator of tendon degradation, yet its genome-scale transcriptional and epigenetic consequences in tenocytes remain incompletely characterized.

**Methods:**

We profiled the transcriptomes and chromatin accessibility landscapes of control and IL-1β-treated primary rat tenocytes using RNA-seq and ATAC-seq, respectively. Differential expression and accessibility analyses, region-associated differentially expressed gene (RAD) analysis, gene functional enrichment analysis, and transcription factor motif enrichment analysis were performed to delineate the regulatory mechanisms underlying IL-1β-induced cellular response.

**Results:**

IL-1β elicited a profound transcriptional alteration, with 1, 468 differentially expressed genes (811 upregulated and 657 downregulated). Upregulated genes were enriched for immune and inflammatory response pathways, whereas downregulated genes encompassed tendon-specific matrix programs and broader mesenchymal developmental pathways. Concordantly, ATAC-seq revealed 15, 257 differentially accessible regions, with a striking asymmetry toward increased accessibility under IL-1β treatment. Integrative analysis revealed a potential association between chromatin remodeling and transcriptional alterations. Motif enrichment within IL-1β-high accessible regions (IL-1β-HARs) identified C/EBP and NF-κB family members as candidate regulators of inflammatory gene activation, while control-HARs (Ctrl-HARs) harbored motifs for homeostatic transcription factors.

**Conclusions:**

These findings demonstrate that IL-1β affects tenocyte homeostasis by simultaneously activates inflammatory gene programs and suppresses tendon-associated expression through coordinated transcriptional and epigenetic remodeling. The transcription factor regulatory elements identified here provide a mechanistic hint for future investigation, and we hypothesize that targeting these elements may offer a potential therapeutic strategy for inflammatory tendon disorders, while this possibility remains to be tested experimentally.

## Introduction

Tendons are dense connective tissues that transmit contractile forces from muscle to bone, enabling locomotion and joint stability. The structural integrity of tendon tissue depends predominantly on tenocytes — specialized fibroblast-like cells embedded within a highly organized extracellular matrix (ECM) rich in type I collagen. Tenocytes regulate ECM homeostasis through the continuous synthesis and remodeling of collagen fibrils, proteoglycans, and glycoproteins, thereby maintaining the mechanical properties essential for tendon function (1).

Tendinopathy represents a common and clinically significant musculoskeletal condition characterized by tendon degeneration, pain, and impaired functional capacity (2). Despite its high prevalence among both athletic and sedentary populations, a clear understanding of the molecular and cellular mechanisms driving tendinopathy remain challenging (2). Historically conceptualized as either an inflammatory or degenerative process, contemporary evidence supports a more nuanced model in which persistent low-grade inflammation and ECM degradation converge to produce a failed healing response. Central to this pathophysiological framework is the pro-inflammatory cytokine interleukin-1β (IL-1β), which functions as a principal mediator of tenocyte dysfunction and tendon matrix degeneration (3–7).

IL-1β is widely recognized as a master regulator of the tenocyte inflammatory response (8, 9). Following binding to the type I IL-1 receptor (IL-1R1) and its co-receptor IL-1RAcP, IL-1β initiates a canonical intracellular signaling cascade through the adaptor protein MyD88, leading to activation of the IκB kinase (IKK) complex and subsequent nuclear translocation of nuclear factor-κB (NF-κB) (10). The study by Tsuzaki et al. established that IL-1β stimulation of human tendon cells potently induces expression of cyclooxygenase-2 (COX-2), matrix metalloproteinases (MMP-1, MMP-3, MMP-13), ADAMTS-4, and additional pro-inflammatory cytokines including IL-6, thereby establishing an auto-amplificatory inflammatory loop that perpetuates catabolic signaling (5). Zhang et al. demonstrated that IL-1β irreversibly inhibits expression of Scx and Tnmd in tendon-derived progenitor cells, effectively blocking their commitment to the tenogenic lineage and redirecting cellular programs toward degenerative phenotypes (6).

While the transcriptional consequences of IL-1β signaling in tenocytes have been extensively characterized, the mechanisms through which this cytokine remodels the chromatin landscape to enable transcriptional reprogramming remain poorly understood. Chromatin accessibility serves as a key determinant of transcriptional responses, as regions of open chromatin permit transcription factor binding and RNA polymerase recruitment at regulatory elements (11). Under inflammatory conditions, stimulus-induced chromatin remodeling creates or eliminates accessible regions that govern the expression of effector genes, thereby shifting transcriptional programs (12). The Assay for Transposase-Accessible Chromatin using sequencing (ATAC-seq) has emerged as a powerful methodology for profiling genome-wide chromatin accessibility and inferring transcription factor activity through motif enrichment analysis (13). In tendon biology, ATAC-seq has been applied to map the chromatin landscape of developing and maturing tendon tissue, revealing dynamic changes in accessible regions that accompany tenocyte differentiation and growth (14). Jones et al. demonstrated that mechanical cues direct tendon cell function at both transcriptional and chromatin accessibility levels, with Yap and Taz serving as key mechanoepigenetic regulators of ECM homeostasis (15). Recently, integrative single-cell RNA-seq and ATAC-seq approaches have uncovered disease-associated chromatin remodeling regulate tendinopathy through FOXO1-PRDX2-TNF axis (16). Single-nucleus transcriptional and chromatin accessibility profiling of developing mouse Achilles tendon has identified Cd55- and Cd248-positive tendon stem/progenitor cells (TSPCs) and revealed their distinct epigenetic architecture, including candidate upstream transcription factors and dynamic accessibility changes during tendon maturation (17). However, these studies have focused either on developmental tendon maturation or on disease-associated chromatin remodeling; the direct effects of inflammatory cytokines on chromatin accessibility and and gene expression in tenocytes remain unexplored.

To investigate whether IL-1β exposure triggers a coordinated remodeling of the tenocyte epigenome and transcriptome, we performed integrated RNA-seq and ATAC-seq profiling of primary rat tenocytes treated with or without IL-1β. We demonstrate that IL-1β induces chromatin remodeling with 15, 257 differential accessible regions (DARs), alongside transcriptional reprogramming encompassing 1468 differentially expressed genes (DEGs). Integration of ATAC-seq and RNA-seq datasets identified concordance between chromatin accessibility and gene expression changes. Motif enrichment analysis of IL-1β-specific accessible regions revealed the transcription factors Cebpb, Cebpd, and NF-κB2 as key candidate regulators of the inflammatory tenocyte response. These findings provide a comprehensive epigenomic map of IL-1β-responsive tenocytes and identify underlying regulatory mechanisms that may help to the treatment for tendinopathy.

## Materials and methods

### Culture and stimulation of tenocytes

Primary tenocytes were isolated from tail tendons of adult male Sprague–Dawley rats. Rats were anesthetized via inhalation of 3% isoflurane in oxygen, and euthanasia was performed by CO_2_ inhalation followed by cervical dislocation as a secondary confirmatory method. Tail tendon tissues were harvested immediately following euthanasia and transferred to ice-cold sterile PBS supplemented with 1% penicillin–streptomycin for subsequent cell isolation. The tendon tissues was digested for 4–6 hours at 37 °C with 0.2% type I collagenase (Gibco, 17100017). The reaction was neutralized with an equal volume of culture medium consisting of low glucose Dulbecco’s modified Eagle’s medium (L-DMEM) (Gibco, USA), 10% (v/v) fetal bovine serum (FBS) and 1% (v/v) penicillin/streptomycin solution. Once the adherent cells reached 80% confluence, they were passaged using 0.25% trypsin (Gibco, 25200056). Cells were cultured at 37 °C in an atmosphere of 5% CO2. Tenocytes were subjected to inflammatory priming upon reaching approximately 70% confluence. The basal culture medium was replaced with fresh medium containing 2 ng/mL IL-1β (Novoprotein, CG93). Following 72 hours of stimulation, cells were collected for transcriptomic and chromatin accessibility profiling. RNA sequencing (RNA-seq) was performed with three independent biological replicates per condition (n = 3), whereas ATAC-seq was conducted with two biological replicates per group (n = 2). Each biological replicate consisted of tenocytes isolated from a separate rat. Cells from different animals were processed independently throughout isolation, culture, cytokine treatment, and downstream processing to ensure biological independence.

### RNA sequencing and data analysis

Total RNA was extracted using a cell RNA isolation kit (Vazyme, RC102). Only samples with an A260/A280 ratio of 1.8-2.2 and RNA integrity number (RIN) >9 were used for library construction. Strand-specific libraries were prepared using the Illumina Stranded mRNA Prep Ligation kit (Illumina, San Diego, CA), and sequenced as 150 bp paired-end reads on the NovaSeq 6000 platform.

Raw reads underwent quality assessment and adapter trimming with fastp (v0.23.2) (18), followed by alignment to the Rattus norvegicus reference genome (rn7) using STAR (v2.7.10a) (19). Gene-level read counts were generated with featureCounts (v2.0.6) (20). Sample reproducibility was evaluated by principal component analysis (PCA) and Euclidean distance heatmaps. Differential expression analysis was performed using DESeq2 (v1.50.2) (21). Differentially expressed genes (DEGs) were identified using a negative binomial generalized linear model with Wald’s test and independent hypothesis weighting for multiple testing correction. Genes with adjusted p < 0.05 and |log2(fold change)| > 1 were considered significant.

### Functional enrichment analysis

Gene Ontology (GO) enrichment analysis of DEGs was conducted using clusterProfiler (v4.6.0) (22). Over-representation analysis was performed separately for upregulated and downregulated genes against the GO Biological Process ontology with Benjamini–Hochberg correction. Gene set enrichment analysis (GSEA) was performed on tendon-related genes (23).

### Quantitative real-time PCR

Quantitative real-time PCR was performed using SYBR Green QPCR Master Mix on a Light Cycler apparatus (Roche 480II). Relative expression values were calculated with the 2^-△△Ct^ method and normalized to *Gapdh* expression.

### ATAC sequencing and data analysis

ATAC-seq libraries were prepared using a high-sensitivity open chromatin profile kit (Novoprotein, N248) following the manufacturer’s instructions. Briefly, 50, 000 cells per sample were lysed, nuclei were transposed with Tn5 transposase at 37 °C for 30 min, and libraries were amplified for 10–12 cycles with saturation monitored by qPCR. Fragments between 150–700 bp were size-selected using AMPure XP beads and sequenced as 150 bp paired-end reads on the NovaSeq 6000. Transposed DNA fragments were subsequently purified and PCR-amplified using indexed adapters. Amplified libraries were then purified using DNA clean beads and sequenced as 150 bp paired-end reads on the Illumina NovaSeq 6000 platform.

Reads were trimmed with fastp (version 0.23.2) and aligned to rn7 using Bowtie2 (v2.4.5) (18, 24). Duplicate reads were removed using picard (http://broadinstitute.github.io/picard). Peaks were called per sample with MACS2 (v2.2.9.1) (25). A consensus peak set was generated by merging all sample peaks, and read counts were quantified for differential accessibility analysis using DESeq2 (v1.50.2) (21). Peaks with adjusted p < 0.05 and |log2(fold change)| > 2 were considered significant. Regions with increased accessibility in IL-1β-treated samples were designated IL-1β-high accessible regions (IL-1β-HARs); those enriched in controls were designated Ctrl-HARs. Genomic distribution of accessible regions relative to genomic features was annotated using Homer (v4.11) (26). Metaplots and heatmaps of RPKM-normalized signal across differentially accessible regions were generated with deepTools (v3.5.5) (27).

### Region associated differentially expressed genes analysis

To assess the cis-regulatory link between chromatin accessibility and transcriptional changes, we performed region associated differentially expressed genes (RAD) analysis (28). The following parameters were specified: rn7 as the reference genome; peak extension distances of 1, 000, 500, 200, 100, 50, 10, 5, 1 and 0 kb; and a hypergeometric test for statistical significance assessment.

### Motif enrichment analysis

Transcription factor motif enrichment in IL-1β-HARs and Ctrl-HARs was performed using Homer (v4.11) findMotifsGenome.pl with default settings (26).

### Statistical analysis

All statistical analyses were conducted in R (v4.5.2). For RNA-seq and ATAC-seq differential analyses, P values were adjusted for multiple comparisons using the Benjamini–Hochberg false discovery rate (FDR) method. For qPCR analysis, data are presented as mean with SD, and Student’s t-test was used to assess the statistical significance between groups.

## Results

### IL-1β induces significant transcriptomic changes in tenocytes

To characterize the transcriptional response of tenocytes to inflammatory stimulation, we first profiled the gene expression of control and IL-1β-treated rat tenocytes by RNA-seq. Principal component analysis (PCA) is a method that reduces complex, high-dimensional data into a few major axes of variation. The first principal component (PC1) captures the largest source of variation in the data, while the second principal component (PC2) captures the next largest source, independently of PC1. In our data, the control and IL-1β-treated samples separated clearly along the PC1, which accounted for 99.3% of the total variance (**Figure 1A**). By contrast, PC2 explained only 0.2% of the variance, confirming that IL-1β treatment was the principal driver of transcriptional variation. Hierarchical clustering of sample-to-sample Euclidean distances corroborated this finding, with control and IL-1β-treated replicates forming distinct, non-overlapping clusters (**Figure 1B**). Together, these metrics established both high intra-group reproducibility and a pronounced IL-1β-driven transcriptional response.

**Figure 1:**
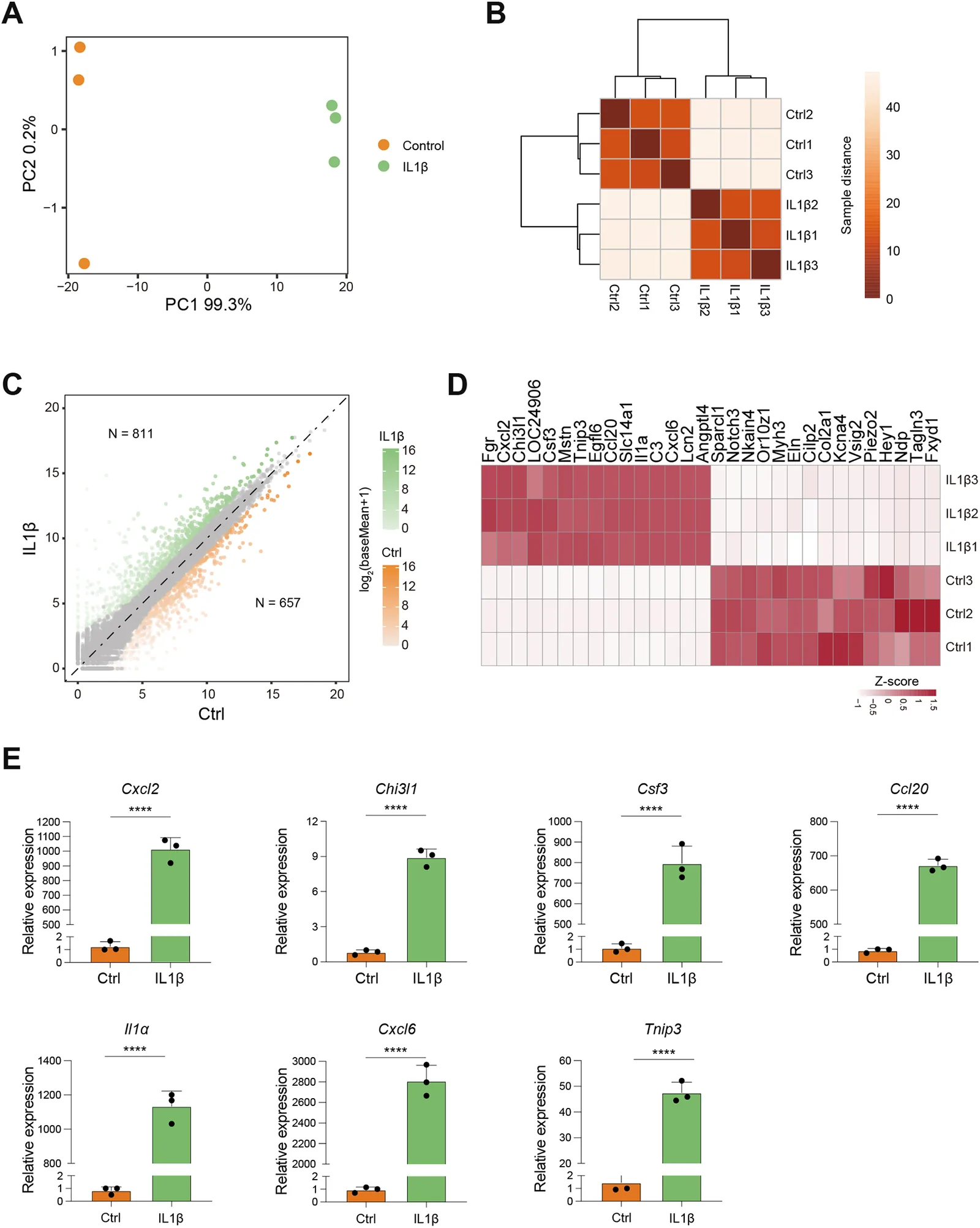
Transcriptomic analysis of IL-1β-treated and control tenocytes. **(A)** Principal component analysis (PCA) of RNA-seq samples from IL-1β-treated and control tenocytes. Each point represents one sample. **(B)** Heatmap plot showing the Euclidean distance between samples from IL-1β-treated and control groups. Darker colors indicate smaller distances (greater similarity between samples), whereas lighter colors indicate larger distances. **(C)** Scatter plot of differentially expressed genes (DEGs) between IL-1β-treated and control groups. N represent the number of highly expressed genes in each group. **(D)** Heatmap plot displaying the expression of top 30 DEGs. Darker red indicates higher relative expression. **(E)** qPCR results showing the expression levels of inflammatory response genes. Bar charts show the mean of three independent biological replicates for each group. *p < 0.05, **p < 0.01, ***p < 0.001, ****p < 0.0001. Data are presented as mean with SD.

Differential expression analysis using DESeq2 identified 1, 468 genes with significantly altered expression under IL-1β stimulation (adjusted p < 0.05, |log2(fold change)| > 1). Of these, 811 genes were upregulated and 657 genes were downregulated in IL-1β-treated tenocytes relative to controls (**Figure 1C**). The scatter plot of mean normalized expression revealed a broad dynamic range of transcriptional changes, with both up- and downregulated genes distributed broadly from low to high abundance (**Figure 1C**).

### DEGs and functional enrichment analysis reveals inflammatory activation and tendon gene suppression upon IL-1β treatment

The top induced genes included key immune mediators such as *Cxcl2*, *Chi3l1*, *Csf3*, *Ccl20*, *Il1α*, and *Cxcl6*, indicating the activation of inflammatory response (**Figures 1D, E**). *Tnip3*, an NF-κB inhibitor-interacting protein, was also markedly upregulated, suggesting the induction of a negative feedback loop that attenuates inflammatory signaling (**Figures 1D, E**). Conversely, the top suppressed genes included established tendon-associated transcripts, such as *Sparcl1*, *Eln*, *Piezo2*, and *Tagln3* (**Figure 1D**) (29–32).

To define the biological processes underlying these transcriptional changes, we performed Gene Ontology (GO) enrichment analysis. Upregulated genes were significantly enriched for immune and inflammatory response pathways (**Figure 2A**). Prominent categories included cellular response to biotic stimulus, cellular response to lipopolysaccharide, regulation of the ERK1/ERK2 cascade, and cellular response to interleukin-1. Chemotaxis-related processes, including leukocyte chemotaxis, myeloid leukocyte migration and cell chemotaxis, were also highly represented. Downregulated genes were enriched for extracellular matrix organization, connective tissue development, and mesenchymal differentiation (**Figure 2B**). The most significant categories included extracellular matrix organization, mesenchymal development. These findings indicate that IL-1β treatment shifts tenocytes away from their homeostatic state, suppressing not only tendon-specific matrix programs but also broader mesenchymal developmental pathways.

**Figure 2:**
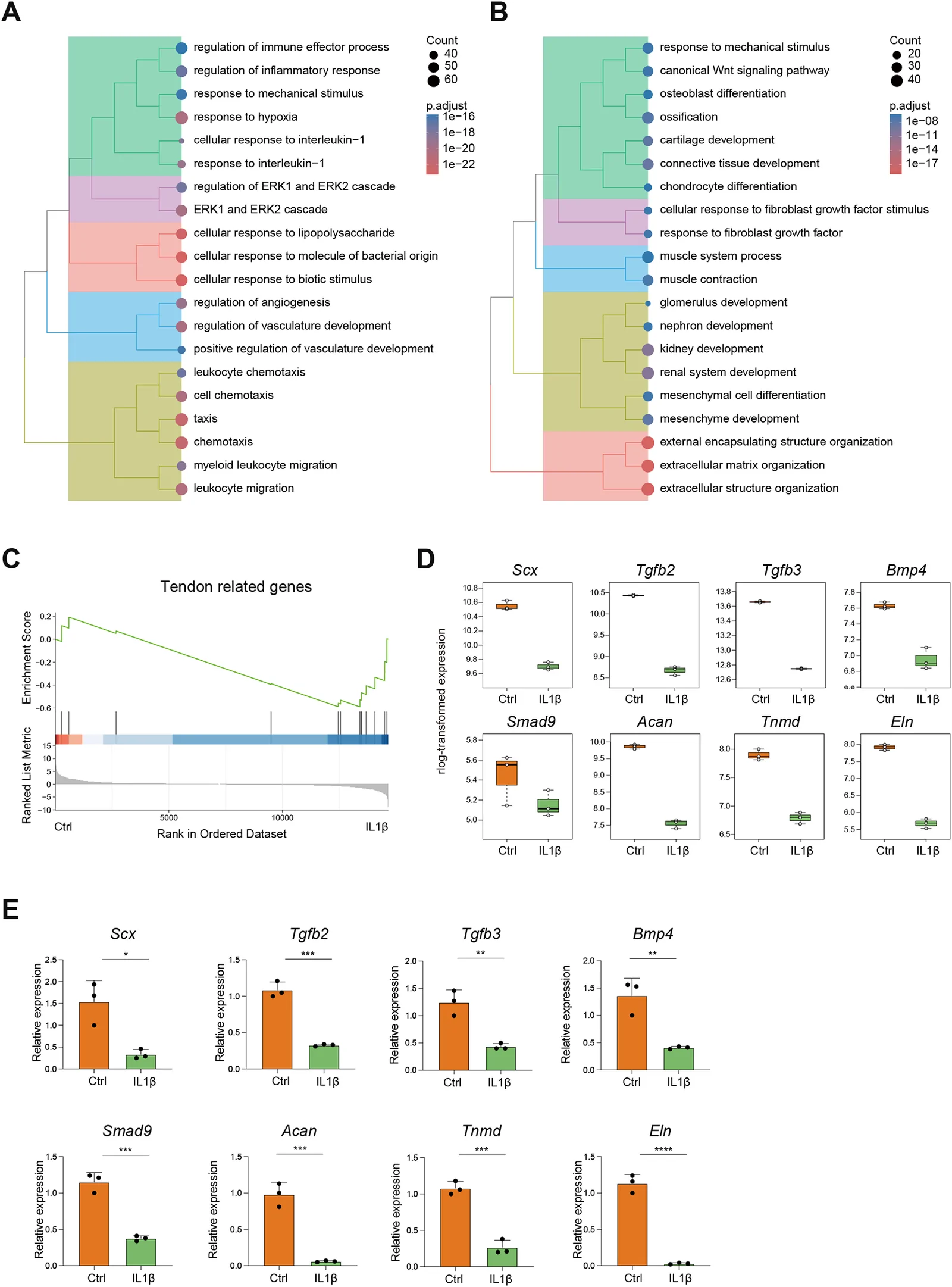
Functional enrichment analysis of differentially expressed genes between IL-1β-treated and control groups. **(A)** Tree plot of gene ontology analysis of genes up-regulated in IL-1β-treated group. Circle size represents the number of enriched genes, and circle color represents the adjusted P value. **(B)** Tree plot of gene ontology analysis of genes down-regulated in IL-1β-treated group. Circle size represents the number of enriched genes, and circle color represents the adjusted P value. **(C)** Gene set enrichment analysis revealing the negative correlation between IL-1β treatment and tendon-related genes. **(D)** Gene expression levels of representative tendon-related genes. **(E)** qPCR results showing the expression levels of tendon-related genes. Bar charts show the mean of three independent biological replicates for each group. *p < 0.05, **p < 0.01, ***p < 0.001, ****p < 0.0001. Data are presented as mean with SD.

We next performed gene set enrichment analysis (GSEA) to evaluate the effect of IL-1β on tendon-related gene expression program. Using a curated set of tendon-associated gene set, GSEA revealed a significant negative enrichment under IL-1β treatment (**Figure 2C**), confirming that IL-1β broadly suppresses the transcriptional identity of tendon cells. To validate this finding, we examined the expression of representative tendon-related genes (**Figures 2D, E**). Both RNA-seq and q-PCR results showed that genes including master tenogenic transcription factor *Scx*, transforming growth factor beta ligands *Tgfb2* and *Tgfb3*, as well as extracellular matrix components *Acan, Tnmd and Eln* were significantly downregulated. Together, these results establish that IL-1β simultaneously activates inflammatory gene programs and suppresses tendon-associated gene expression, driving a functional switch in tenocyte identity.

### IL-1β stimulation induces widespread chromatin remodeling

To determine whether the observed transcriptional reprogramming was accompanied by changes in chromatin accessibility, we profiled the chromatin landscape by ATAC-seq. ATAC-seq peaks showed a consistent genomic distribution across all samples, with the majority mapping to distal intergenic and intronic regions (**Figure 3A**). This distribution is characteristic of open chromatin profiling and confirms the high quality of our accessibility data.

**Figure 3:**
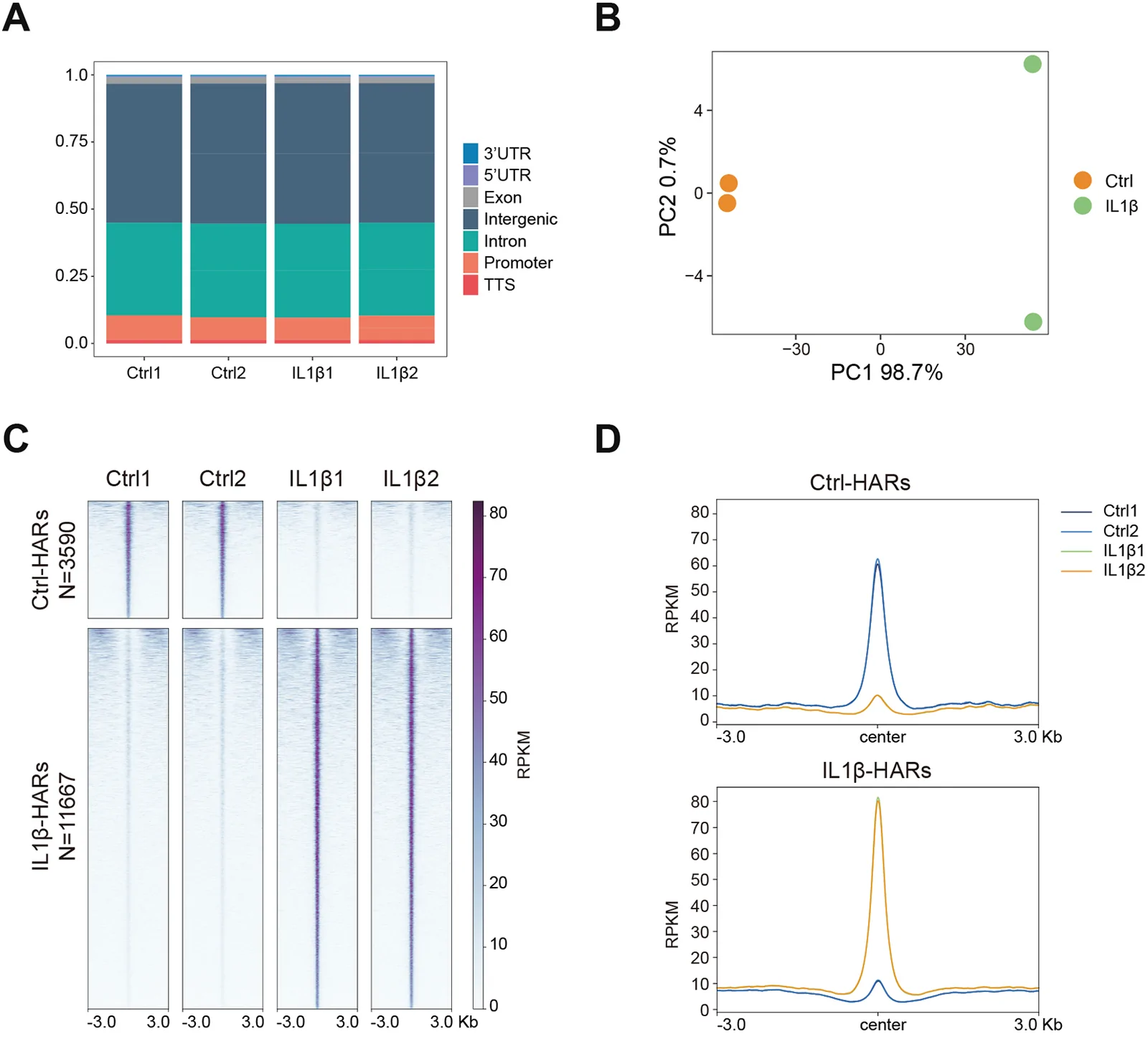
Chromatin accessibility analysis of IL-1β-treated and control tenocytes. **(A)** Stack bar plot showing the genomic distribution of ATAC-seq peaks across distinct genomic features. **(B)** Principal component analysis (PCA) of samples from IL-1β-treated and control tenocytes. Each point represents one sample. **(C)** Heatmap plot showing differential accessible chromatin regions between IL-1β-treated and control groups. Purple represents the reads per kilobase of bin per million of reads sequenced (RPKM) value of ATAC-seq peaks, with darker colors indicating higher values. **(D)** Metaplots of differential accessible chromatin regions between IL-1β-treated and control groups. RPKM: Reads per kilobase of bin per million of reads sequenced.

PCA plot of the ATAC-seq samples showed a clear separation of control and IL-1β-treated conditions, with PC1 explaining 98.7% of the variance in chromatin accessibility (**Figure 3B**). PC2 accounted for merely 0.7% of the variance. These results indicate that IL-1β drives a dominant and reproducible shift in the chromatin accessibility landscape, mirroring the transcriptional response. Differential accessibility analysis identified 15, 257 differential accessibile regions (DARs), of which 11, 667 exhibited increased accessibility in IL-1β-treated tenocytes (IL-1β-high accessible regions, IL-1β-HARs), and 3, 590 showed higher accessibility in control cells (Control-high accessible regions, Ctrl-HARs) (**Figure 3C**). Metaplot of these differentially accessible regions showed that control samples exhibited a sharp peak of accessibility centered on the region midpoint of Ctrl-HARs, whereas IL-1β-treated samples showed substantially reduced signal (**Figure 3D**). Conversely, at IL-1β-HARs, IL-1β-treated samples displayed a prominent accessibility peak and control samples showed only baseline signal (**Figure 3D**).

### Integrated analysis reveals coordinated epigenomic-transcriptomic changes

To determine whether chromatin accessibility changes were functionally linked to transcriptional alterations, region-associated differentially expressed gene (RAD) analysis was performed. IL-1β-HARs showed significant enrichment for association with upregulated genes, with observed-to-expected ratios exceeding 1.0 across proximal and distal distance bins, and the strongest enrichment observed for genes located within 1Kb to the transcription start site (TSS) (**Figure 4A**). Conversely, Ctrl-HARs were significantly associated with downregulated genes (**Figure 4B**). Genome browser visualization at representative loci confirmed the observation. At the *Elf3* and *Mmp10* loci, IL-1β treatment induced both increased chromatin accessibility at the promoter and elevated gene expression (**Figures 4C, D**). Conversely, *Postn* and *Enpp1* displayed strong accessible peaks in control samples that were substantially diminished under IL-1β treatment, paralleling the down-regulated expression of these genes (**Figures 4E, F**). The changes of expression levels of these genes were verified by qPCR analysis (**Figure 4G**).

**Figure 4:**
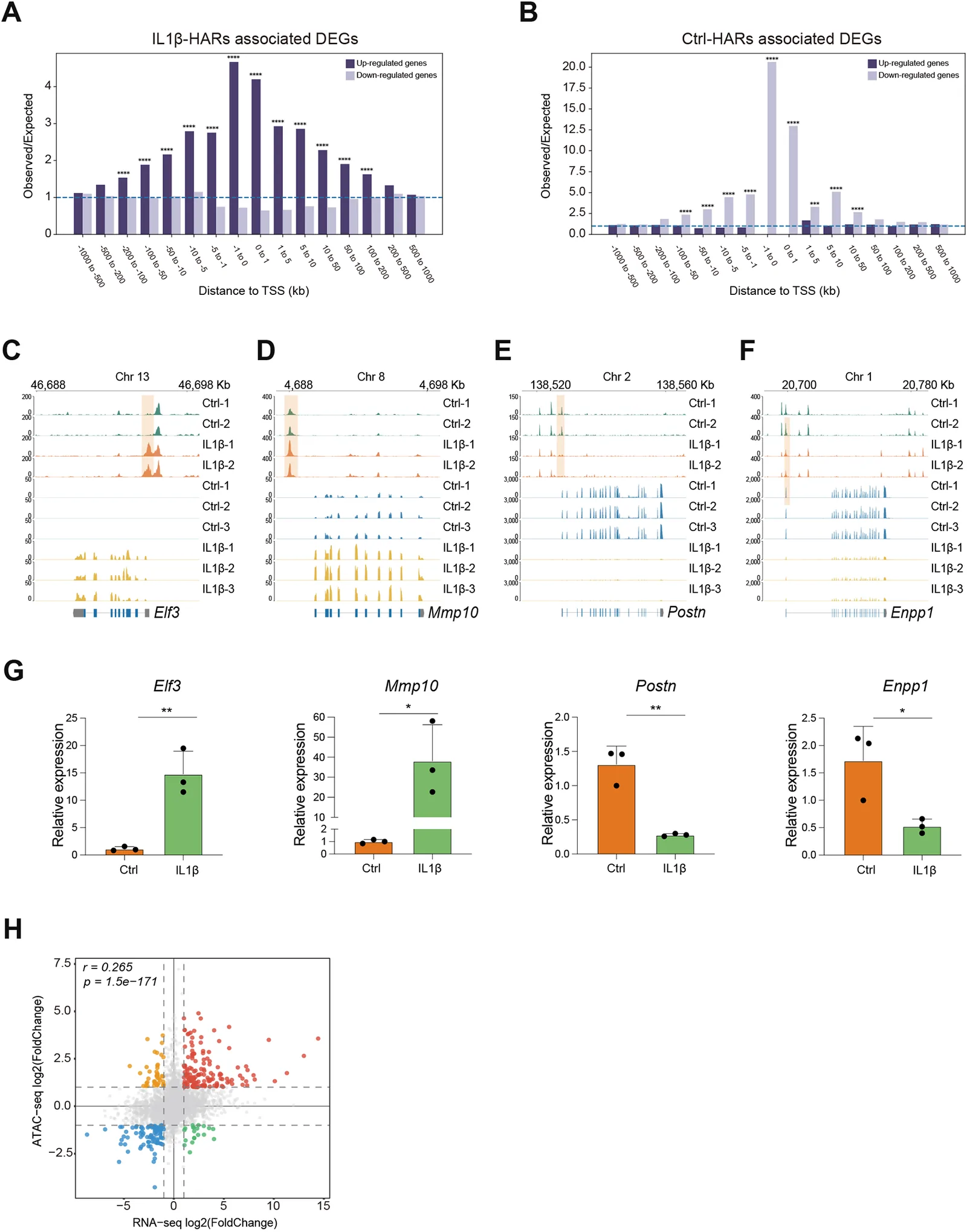
Correlation analysis between differentially accessible regions and DEGs. **(A)** Region-associated differentially expressed gene (RAD) analysis for the association between IL-1β-high accessible regions (IL-1β-HARs) and DEGs. ****P < 0.0001, hypergeometric test. **(B)** RAD analysis for the association between control-high accessible regions (Ctrl-HARs) and DEGs. ****P < 0.0001, hypergeometric test. **(C–F)** Genome browser tracks illustrating the ATAC-seq signal and gene expression profiles of *Elf3*, *Mmp10*, *Postn* and *Enpp1*. The top four tracks show ATAC-seq signal, and the bottom six tracks show RNA-seq signal. The y-axis indicates RPKM. **(G)** qPCR results showing the expression levels of *Elf3*, *Mmp10*, *Postn* and *Enpp1*. Bar charts show the mean of three independent biological replicates for each group. *p < 0.05, **p < 0.01. Data are presented as mean with SD. **(H)** Scatter plot showing concordance between transcriptional and chromatin accessibility changes.

We also examined the correlation of fold changes between DEGs and DARs within TSS. This analysis revealed a significant positive correlation (r = 0.265, p = 1.5 × 10^-171^; **Figure 4H**). Notably, the correlation coefficient reflects moderate effect size, indicating that DARs within TSS only accounts for a proportion of gene expression changes, the rest of genes may be affected by distal regulatory elements.

### IL-1β stimulation remodel transcription factor binding activity

To identify the transcription factors driving IL-1β-induced chromatin remodeling, we performed motif enrichment analysis using Homer on IL-1β-HARs and Ctrl-HARs separately. Among IL-1β-HARs, the most enriched motifs included the CEBP and NF-κB, reflecting established roles for these signaling in IL-1β responses (**Figure 5A**). Concordantly, genome browser tracks and q-PCR results confirmed upregulation of the C/EBP family members *Cebpb* and *Cebpd*, as well as the *Nfkb2*, following IL-1β stimulation (**Figures 5B, C**). Conversely, Ctrl-HARs were enriched for motifs recognized by transcription factors associated with mesenchymal and tendon homeostatic functions (**Figure 5D**). The enrichment of these motifs in control-biased accessible regions raises the possibility that IL-1β treatment reduces the accessibility of regions may bound by homeostatic transcription factors. This reduced accessibility could contribute to the coordinated suppression of tendon-related gene expression observed in our transcriptomic analysis.

**Figure 5:**
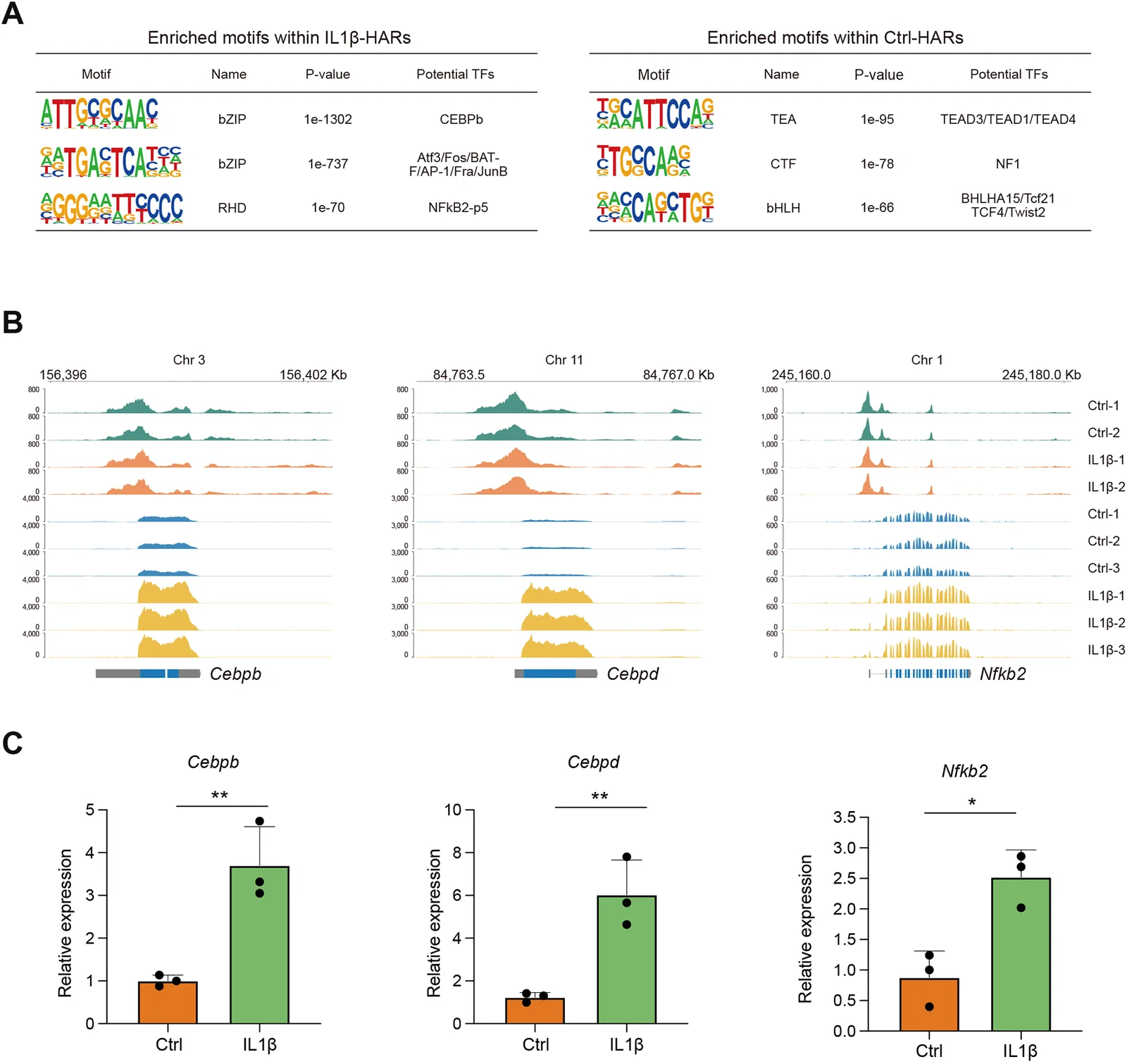
Transcription factor motif enrichment analysis. **(A)** Homer motif enrichment analysis of IL-1β-HARs and Ctrl-HARs. **(B)** Genome browser tracks illustrating the gene expression profiles of *Cebpb, Cebpd* and *Nfkb2* genes. The top four tracks show ATAC-seq signal, and the bottom six tracks show RNA-seq signal. The y-axis indicates RPKM. **(C)** qPCR results showing the expression levels of *Cebpb*, *Cebpd* and *Nfkb2*. Bar charts show the mean of three independent biological replicates for each group. *p < 0.05, **p < 0.01. Data are presented as mean with SD.

## Discussion

In this study, we present the integrated transcriptomic and chromatin accessibility analysis of IL-1β-treated primary rat tenocytes. Our findings establish a molecular framework linking IL-1β signaling to epigenetic remodeling of genes related to inflammatory activation and lineage-specific genes suppression in tendon cells.

Tendon disorders, including tendinopathy and rotator cuff degeneration, are frequently associated with chronic low-grade inflammation driven by pro-inflammatory cytokines, with IL-1β stands as a central mediator (2, 8, 9). Here, we demonstrate that IL-1β elicits a profound and highly consistent transcriptional reprogramming in rat tenocytes, involving 1, 468 differentially expressed genes. The upregulated genes were dominated by established inflammatory mediators, including chemokines (*Cxcl2*, *Ccl20*, *Cxcl6*), colony-stimulating factors (*Csf3*), and the alarmins *Chi3l1* and *Il1α*. These findings align with the well-documented capacity of IL-1β to activate NF-κB and MAPK signaling cascades, driving the production of chemoattractant molecules that recruit immune cells to injured tendon tissue (33, 34). Notably, the concomitant induction of *Tnip3*, an NF-κB inhibitor-interacting protein, suggests the parallel activation of negative feedback circuits that may serve to limit excessive inflammatory signaling. Whether this feedback mechanism is sufficient to protect against chronic IL-1β-driven tendon degeneration *in vivo* warrants further investigation, as prolonged inflammatory signaling has been implicated in failed tendon healing (35). Equally significant was the broad suppression of tendon-associated genes under IL-1β treatment. The downregulation of Scx is particularly noteworthy given its central role in maintaining tenocyte fate and tendon matrix integrity during development and adult homeostasis (36–38). These observations are consistent with previous reports that inflammatory cytokines compromise tenogenic differentiation, but our analysis extends this understanding by revealing the systematic dismantling of the entire tendon transcriptional identity program rather than isolated gene suppression (39). Collectively, the simultaneous activation of inflammatory gene networks and suppression of tendon-specific transcriptional programs establishes IL-1β as a destabilizer of tenocyte identity.

Our ATAC-seq analysis reveals that IL-1β induces reorganization of the chromatin accessibility landscape, with over 15, 000 differentially accessible regions and a striking asymmetry: approximately 76% of DARs exhibited increased accessibility (IL-1β-HARs), while only 24% showed reduced accessibility (Ctrl-HARs). This asymmetry suggests that IL-1β predominantly acts through the opening of new regulatory elements rather than the closing of existing ones, a pattern consistent with an active, cytokine-driven remodeling process. The central question motivating our multi-omics approach was whether chromatin accessibility changes are associated with the observed transcriptional alterations. Our RAD analysis provides evidence for such link: IL-1β-HARs were significantly enriched for association with upregulated genes, while Ctrl-HARs were associated with downregulated genes. This reciprocal relationship suggests a link between cytokine-induced chromatin opening and transcriptional activation, as well as between chromatin closure and transcriptional suppression (13, 40). The positive correlation between DEG and DAR fold changes at TSS regions further supports this association, although the moderate correlation coefficient indicates that TSS-proximal accessibility changes account for only a subset of transcriptional variation. This observation carries important mechanistic implications: the majority of IL-1β-induced expression changes are likely governed by distal regulatory elements—enhancers and silencers that act over long genomic distances through chromatin looping. To support this interpretation, it’s needed to examine regulatory architecture at genome-wide scales using techniques such as Hi-C (41).

To understand the upstream regulatory factors governing IL-1β-induced chromatin remodeling, we performed transcription factor motif enrichment analysis. The most significantly enriched motifs within IL-1β-HARs included binding sites for C/EBP family members and the NF-κB subunit p52 (also named NF-κB2). C/EBP transcription factors are well-established mediators of IL-1β and LPS responses, orchestrating the expression of pro-inflammatory cytokines and chemokines through the activation of enhancer elements (42, 43). NF-κB2, a component of the non-canonical NF-κB pathway, has been increasingly implicated in sustaining chronic inflammatory states—a feature particularly relevant to the persistent low-grade inflammation characteristic of tendinopathy (44). The most significantly enriched motifs for Ctrl-HARs belong to TEA transcription factor family, including TEAD1, TEAD3 and TEAD4. TEAD factors are central mediators of YAP/TAZ-dependent mechanotransduction, the pathway through which tenocytes sense and respond to mechanical loading (14, 45). Reduced accessibility at regions harboring homeostatic transcription factor motifs under IL-1β treatment may help explain the observed loss of tendon gene expression. One plausible model is that, as inflammatory transcription factors gain access to newly opened chromatin regions, homeostatic transcription factors lose access to their binding sites, leading to a shift in the regulatory elements that defines tenocyte identity. However, this model remains speculative and requires direct experimental validation, such as TF perturbation or binding assays.

In this study, tenocytes were stimulated with 2 ng/mL IL-1β for 72 h. This concentration was selected based on previous *in vitro* tendinopathy models, in which IL-1β is commonly used at 1–17 ng/mL to stimulate tenocytes and tendon-derived cells and consistently induces changes in tendon cell gene expression (6, 46–48). In addition, a recent study examining the inflammatory cytokine profile in tendon ultrafiltrate after tendon injury reported that IL-1β peaked at 48 h post-injury, with a median concentration of 2, 097 pg/mL (range 668–5, 505 pg/mL) (49). The concentration used here therefore approximates the *in vivo* range reported after tendon injury while reliably inducing inflammatory responses in tenocytes. The 72 hours stimulation period was chosen to capture the transition from acute inflammatory signaling to more sustained transcriptional reprogramming, which is relevant to the early phases of chronic tendinopathy, a condition characterized by sustained low-grade inflammation, elevated pro-inflammatory cytokines, and progressive loss of tendon-specific matrix gene expression. Our findings that IL-1β simultaneously activates inflammatory gene programs and suppresses tendon-associated genes through coordinated epigenetic remodeling provide mechanistic insights into this transition. However, this *in vitro* model does not replicate the complex *in vivo* microenvironment, and additional time points and *in vivo* tendinopathy models may be needed to determine how the chromatin accessibility and gene expression changes over time.

Several other limitations of this study should also be acknowledged. First, our experiments were conducted using primary rat tenocytes in two-dimensional monolayer culture, which may not fully recapitulate the complex three-dimensional extracellular matrix environment and mechanical loading conditions that tenocytes experience *in vivo*. Second, our bulk sequencing approach averages signals across the entire cell population, masking potential cell-to-cell heterogeneity in the IL-1β response. Single-cell multi-omics approaches would reveal whether all tenocytes respond uniformly to IL-1β or whether subpopulations with distinct responsiveness exist. Third, while our integrative analysis reveals a correlation between chromatin accessibility and gene expression changes, functional validation through CRISPR-mediated enhancer perturbation or transcription factor ChIP-seq is required to establish causal relationships.

## Conclusions

In summary, this study establishes IL-1β as a potent inducer of coordinated transcriptional and epigenetic remodeling in tenocytes. Through multi-omics integration, we demonstrate that IL-1β simultaneously activates inflammatory gene programs driven by C/EBP and NF-κB transcription factors, and suppresses tendon-specific gene expression by reducing chromatin accessibility at regions containing motifs of homeostatic transcription factors. These findings advance our understanding of tendinopathy pathogenesis by showing that, in cultured tenocytes, IL-1β induces coordinated transcriptomic and chromatin accessibility changes consistent with a shift away from the tendon-associated transcriptional program, although whether this reflects a reversible or irreversible loss of tenocyte identity *in vivo* remains to be determined. The identification of specific transcription factors and regulatory elements involved in this process provides a clue for future studies, and we speculate that these factors and elements may represent candidate targets for therapeutic strategies aimed at preserving tenocyte function in inflammatory tendon disorders. Validation of this hypothesis will require TF perturbation, rescue experiments, and *in vivo* tendinopathy models.

## Data Availability

The datasets presented in this study can be found in online repositories. The names of the repository/repositories and accession number(s) can be found below: PRJCA068523 (https://ngdc.cncb.ac.cn/bioproject/browse/PRJCA068523).
